# Second Class Postal Rates

**Published:** 1912-11

**Authors:** 


### Second Class Postal Rates

As we have stated before, this Journal does not ask charity from the Postoffice, any more than from anyone else. While we believe that periodical literature is, on the whole, a great educational factor in modern life, there can be no question but that the multiplication of certain kinds of magazines tends to an enormous waste of time. Mere reading is not study. Nor would it be practicable to frame laws or to interpret them if framed, so as to discriminate between educative and non-educative periodicals.

But we do believe that the Postoffice Department should give the lowest rates compatible with self-support and that the rates on periodicals should be based on ordinary principles of wholesale as opposed to retail business, on the fact that such mail does not require the personal attention requisite for letters, that time may be saved in handling it because slight delays make no material difference with the interests of publishers and recipients, that except for the calories of locomotives, horses and men in hauling and transporting, it costs no more to deliver a half pound magazine than an ounce letter. It should also be realized that the circulation of magazines involves an enormous amount of first class correspondence, and that the maintenance of the latter should be allowed for in the rate charged for second class mail.

The P. M. G. estimates that second class matter actually costs the department 9 2-3 cents per pound. Any material increase in such mail, should, therefore, lead to an enormous deficit. The facts are as follows: (Overstreet Postal Commission.)

| | Weight of (Paid) 2d Class Matter | Deficits. | Surplus. |
|---|---|---|---|
| 1907 | 712,945,176 | $ 6,653,283.00 | |
| 1908 | 694,865,884 | 16,873,223.00 | |
| 1909 | 723,233,182 | 17,441,719.00 | |
| 1910 | 817,428,141 | 5,848,561.88 | |
| 1911 | 893,309,893 | | $219,118.12 |

The parcels post which goes into operation January 1st, next, will be of great benefit to doctors. The following are the rates established:

| | First Pound | Each Additional Pound | Eleven Pounds |
|---|---|---|---|
| Rural route and city delivery | .05 | .01 | .15 |
| 50 mile zone | .05 | .03 | .35 |
| 150 mile zone | .06 | .04 | .46 |
| 300 mile zone | .07 | .05 | .57 |
| 600 mile zone | .08 | .06 | .68 |
| 1000 mile zone | .09 | .07 | .79 |
| 1400 mile zone | .10 | .09 | 1.00 |
| 1800 mile zone | .11 | .10 | 1.11 |
| Over 1800 miles | .12 | .12 | 1 32 |

